# HNF1B-mediated repression of SLUG is suppressed by EZH2 in aggressive prostate cancer

**DOI:** 10.1038/s41388-019-1065-2

**Published:** 2019-10-21

**Authors:** Jianqing Wang, Chenxi He, Peng Gao, Siqing Wang, Ruitu Lv, Huihui Zhou, Qidong Zhou, Ke Zhang, Jian Sun, Caibin Fan, Guanxiong Ding, Fei Lan

**Affiliations:** 1 Department of Urology, The Affiliated Suzhou Hospital of Nanjing Medical University, 215002 Suzhou, Jiangsu China; 2 Key Laboratory of Epigenetics and Metabolism, Ministry of Science and Technology, Institutes of Biomedical Sciences; Key Laboratory of Carcinogenesis and Cancer Invasion, Ministry of Education, Liver Cancer Institute, Zhongshan Hospital, Fudan University, 200032 Shanghai, China; 3 Department of Urology, Huashan Hospital, Fudan University, Shanghai, China; 4 Department of pathology, Affiliated Yuhuangding Hospital of Qingdao University, 266071 Shandong, China

**Keywords:** Prostate cancer, Oncogenes

## Abstract

Prostate cancer is the most common malignancy in men in developed countries. Overexpression of enhancer of zeste homolog 2 (EZH2), the major histone H3 lysine 27 methyltransferase, has been connected to prostate cancer malignancy. However, its downstream genes and pathways have not been well established. Here, we show tumor suppressor Hepatocyte Nuclear Factor 1β (HNF1B) as a direct downstream target of EZH2. EZH2 binds *HNF1B* locus and suppresses HNF1B expression in prostate cancer cell lines, which is further supported by the reverse correlation between EZH2 and HNF1B expression in clinical samples. Consistently, restored HNF1B expression significantly suppresses EZH2-mediated overgrowth and EMT processes, including migration and invasion of prostate cancer cell lines. Mechanistically, we find that HNF1B primarily binds the promoters of thousands of target genes, and differentially regulates the expression of 876 genes. We also identify RBBP7/RbAP46 as a HNF1B interacting protein which is required for HNF1B-mediated repression of SLUG expression and EMT process. Importantly, we find that higher HNF1B expression strongly predicts better prognosis of prostate cancer, alone or together with lower EZH2 expression. Taken together, we have established a previously underappreciated axis of EZH2-HNF1B-SLUG in prostate cancer, and also provide evidence supporting HNF1B as a potential prognosis marker for metastatic prostate cancer.

## Introduction

Prostate cancer is one of the leading causes of cancer-related mortality in men, particularly in developed countries [1]. Metastatic prostate cancer, especially the metastatic castration-resistant prostate cancer, is the lethal stage of prostate cancer, which is the major catalyst of morbidity and mortality in the patients [2]. Although extensively studied, the underlying mechanism is still not fully understood. Clinically, prognostic markers for different subtypes of prostate cancer are still lacking.

Recent studies revealed critical roles of epigenetic regulation by histone H3 lysine 27 trimethylation (H3K27me3) and DNA methylation, during the tumorigenesis of prostate cancer [3]. Enhancer of zeste homolog 2 (EZH2) is a critical component of the polycomb repressive complex 2 (PRC2) mediating gene silencing mainly through catalyzing histone H3K27me3 at the repressive chromatin regions [4]. EZH2 expression is significantly elevated in prostate cancer and is accompanied with accelerated proliferation and enhanced metastasis capability [5–8]. Recent researches have also confirmed that EZH2 plays an important role in the advanced stages of prostate cancer, including CRPC [2] and neuroendocrine prostate cancer (NEPC) [9]. Importantly, inhibition of EZH2 could significantly suppress prostate cancer cell proliferation and invasion in vivo and in vitro, supporting EZH2 as a promising therapeutic target [10–12]. Despite the important role of EZH2 in prostate cancer development, the downstream genes, and pathways mediating EZH2 oncogenic effect are still not fully revealed, impeding the development of targeted therapeutics.

Hepatocyte nuclear factor 1β (HNF1B), a transcription factor, was originally reported to regulate the Notch pathway components of Lfng, Dll1 and Jag1 and the Irx1/2 factors in ureteric bud branching and the initiation of nephrogenesis [13, 14]. Recent years, genome-wide association studies (GWAS) and fine-mapping analyses had identified several SNP variants within HNF1B gene associated with hyper promoter DNA methylation, reduced HNF1B expression, and increased risk of prostate cancer [15–21], indicating a tumor suppressive role of HNF1B in prostate cancer development. Consistently, ectopically expression of HNF1B in prostate cancer cell lines, PC-3 and DU145, but not normal prostate cell line PNT2a, led to significantly reduced proliferation and paxillin associated adhesion reflecting a reduced Epithelial–mesenchymal transition (EMT) ability [22]. However, whether HNF1B could be suppressed by EZH2 and H3K27me3, and how HNF1B regulates its downstream genes are still unclear.

EMT is a complex biological process in which both genetic and epigenetic events cause epithelial cells to acquire mesenchymal features. This process occurs in normal development and is often hijacked by cancer cells during migration and metastasis [23, 24]. Many factors involved in EMT process are reported [25]. Among those, SLUG/SNAI2, a member of the snail family of C2H2-type zinc finger transcription factors [26], promotes EMT through repressing E-cadherin expression [27]. However, how the EMT process contributes to prostate cancer progression, especially in the context of EZH2 overexpression, remains elusive.

Here, we report a previously unacknowledged axis of EZH2-HNF1B-SLUG in prostate cancer development. We found that HNF1B could be epigenetically repressed by EZH2 in prostate cancer cell lines, and its expression is reversely correlated with EZH2 expression in several cohorts of prostate cancer samples, including TCGA database and in-house collection. Lower HIF1B expression with higher EZH2 is associated with metastases and significantly poorer prognoses. Mechanistically, HNF1B interacts with RBBP7, a component of multiple transcription repressor complexes, and represses SLUG expression and EMT process to suppress the cancer phenotypes of prostate cancer cells.

## Results

### EZH2 binds the first intron of *HNF1B* and suppresses *HNF1B* expression in prostate cancer

**Fig. 1:**
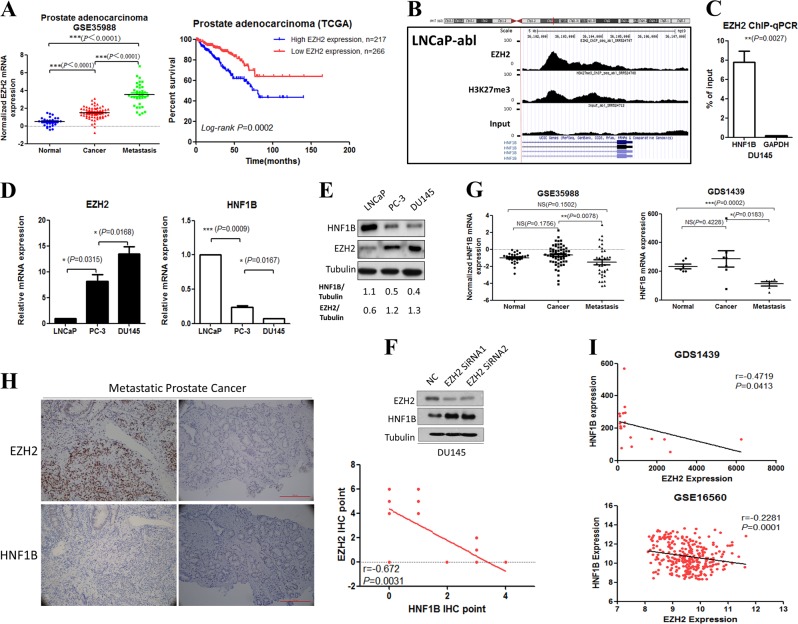
EZH2 binds and represses HNF1B expression in prostate cancer. **a** Association analyses between EZH2 expression and prostate cancer severity and prognoses. **b** EZH2 ChIP-seq signals in HNF1B gene locus. **c** EZH2 binding of HNF1B gene examined by ChIP-qPCR. **d** mRNA levels of EZH2 and HNF1B in metastatic (DU145, PC-3) and nonmetastatic (LNCaP) prostate cancer cell lines. **e** Representative western blot analyses of EZH2 and HNF1B in the indicated prostate cancer cell lines. **f** Western blot analyses of the HNF1B protein in DU145 with the indicated treatments. Tubulin expression was used as a loading control. **g** Expression analyses of HNF1B mRNA level in benign, primary, and metastatic prostate cancer samples. **h** Representative IHC staining of EZH2 and HNF1B protein levels in metastatic prostate cancer tissue. **i** Expression correlation analyses of EZH2 and HNF1B mRNA levels in prostate cancer samples from GDS1439 and GSE16560

Accumulating evidence has shown that EZH2 expression level correlates with the advanced stages of prostate cancer progression and poor prognosis, and its oncogenic function is required for prostate tumorigenesis [28]. To confirm previous findings, we analyzed the microarray data of GSE21032, GSE16560, and GSE35988, and found that the EZH2 mRNA levels in metastatic prostate cancer samples were much higher compared with those of the nonmetastatic prostate cancer samples and the paired normal tissues. More importantly, we found that patients with higher EZH2 expression were associated with significantly poorer prognoses (Figs. 1a and S1A).

To identify the downstream target(s) of EZH2 involved in prostate cancer progression, we explored the public EZH2 and histone H3K27me3 ChIP-seq (chromatin immunoprecipitation assays with sequencing) datasets of various prostate cancer cell lines [29]. We noticed the binding events of EZH2 and histone H3K27me3 in the first intron of *HNF1B*, in LNCaP-abl (Fig. 1b) and VCaP cells (data not shown). Such binding events of EZH2 were then confirmed by ChIP-qPCR in another prostate cancer cell line, DU145 (Fig. 1c). HNF1B is a transcription factor with tumor suppressive function, and our data indicate that it might be suppressed by the EZH2-mediated histone H3K27me3 pathway, in addition to previously reported DNA methylation [22]. Next, we examined HNF1B and EZH2 expression in prostate cancer cell lines with different metastatic potentials. Both mRNA and protein levels of EZH2 were high accompanied with low HNF1B-expressing in the metastatic prostate cancer cell lines PC-3 and DU145. While the nonmetastatic LNCaP cells showed low EZH2 and high HNF1B expression (Fig. 1d, e). Importantly, we further demonstrated that knockdown of EZH2 by RNAi resulted in significant elevated HNF1B expression at both the mRNA (data not shown) and protein levels in PC-3 and DU145 (Fig. 1f), establishing a causative connection.

To further validate the pathological relevance of such connection, we set out to determine the HNF1B expression pattern, and its relationship with EZH2 level in prostate cancer samples. Microarray based expression analyses using GSE35988 and GDS1439 datasets indicated significant reduced HNF1B expression in metastatic prostate cancer samples (Fig. 1g). Significant reverse correlation between HNF1B and EZH2 protein levels was also readily identified by IHC analyses in 17 cases of in-house collection of metastatic prostate cancer samples (*r* = −0.672; *P* = 0.031; Fig. 1h). Supporting this, significant reverse correlation of HNF1B and EZH2 mRNA levels were also confirmed in GDS1439 and GSE16560 datasets (Fig. 1i).

Taken together, our findings established a previously unknown connection between HNF1B and EZH2 in prostate cancer samples and cell lines.

### HNF1B antagonizes the oncogenic activity of EZH2 in prostate cancer cells

**Fig. 2:**
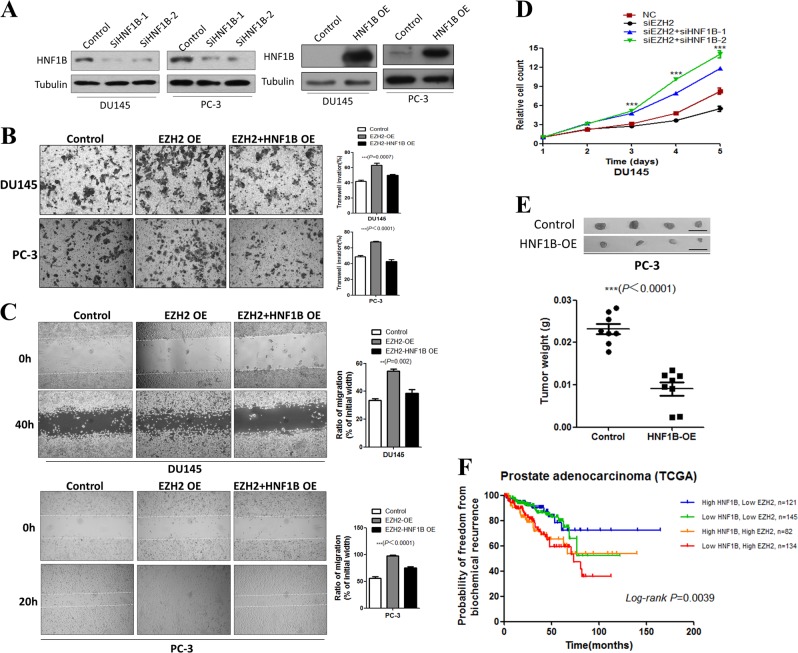
HNF1B suppresses the oncogenic activity of EZH2 in prostate cancer cells. **a** Western blot analyses of HNF1B expression in the indicated cell lines with either overexpression of RNAi knockdown. **b** Transwell assay analyses of the indicated cell lines (scale bars = 100 μm). **c** Wound-healing assay analyses of the DU145 and PC-3 cells transfected with control vector, EZH2, or EZH2 together with HNF1B-expressing vectors. Images were taken at the indicated time points. **d** Cell proliferation was measured at the indicated time points (*P < 0.05, **P < 0.01, ***P < 0.001). **e** Xenograft analyses of PC-3-derived tumors with control vector or HNF1B overexpression (Scale bar, 1 cm, **P < 0.01, ***P < 0.001). **f** High EZH2 and low HNF1B mRNA levels were associated with prostate cancer poor prognoses. A statistically significant increase in RFS was observed in patients with high HNF1B/EZH2 expression ratios (*P* = 0.0039)

It has been reported that EZH2 promotes prostate cancer progression and metastasis via facilitating cell growth, proliferation, and migration [5]. We thus wondered whether HNF1B could play any antagonizing roles in EZH2-mediated oncogenic activities. To test the idea, we first established EZH2 overexpression in DU145 and PC-3 cell lines, which significantly promoted cell migration in the transwell assay, consistent with a previous report [30]. We then ectopically overexpressed HNF1B in the two EZH2 overexpression stable cell lines, and found that the enhanced migration activities by EZH2 overexpression were greatly suppressed (Fig. 2a, b). Similarly, wound-healing assays also showed that HNF1B overexpression could ablate the enhanced migration by EZH2 overexpression (Fig. 2c). In line with the above overexpression studies, we also found that siRNA-mediated HNF1B knockdown could partially rescue the reduced proliferation caused by EZH2 loss in DU145 cells (Fig. 2d). Importantly, such effect is not only limited to 2D culture, as HNF1B overexpression led to significantly retarded growth of PC-3 cells in the SC xenograft assay (Fig. 2e).

Supporting our mechanistic findings, Kaplan–Meier analyses of the TCGA data revealed that high EZH2 and low HNF1B expression were associated with a substantial increase of the relapse frequency while high HNF1B and low EZH2 showed the best prognoses (Fig. 2f), pinpointing the clinical relevance of our findings of EZH2 acts as an oncogenic player at least partially through suppression of HNF1B in prostate cancer.

### HNF1B primarily binds the promoter regions of its target genes

**Fig. 3:**
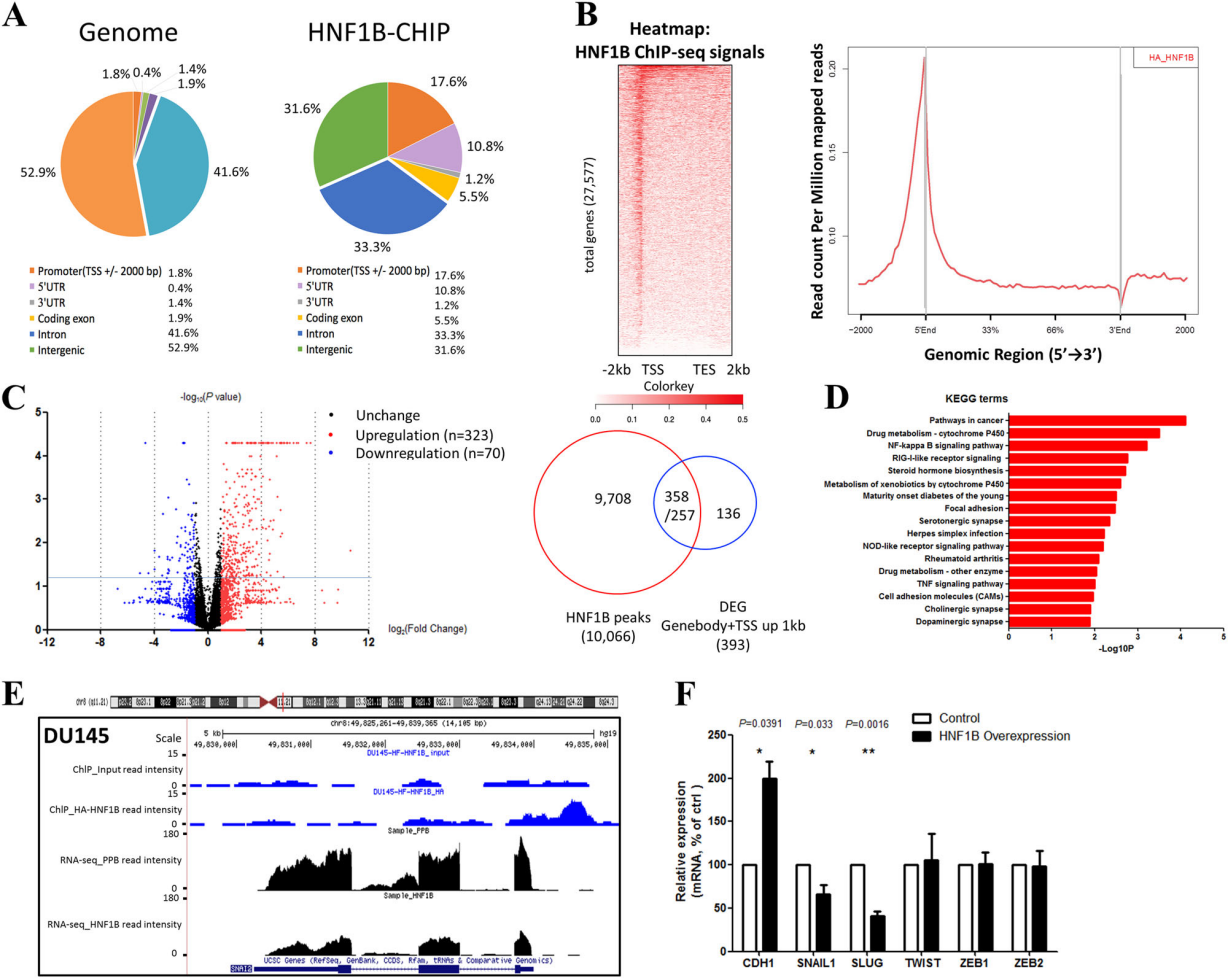
HNF1B binds the promoters and regulates the expression of its target genes in DU145 cell. **a** Genome distribution of HA-HNF1B ChIP-seq peaks in DU145 cells. **b** Heatmap and signal plot of HA-HNF1B ChIP-seq signals in DU145 cells. Heatmap was ranked by HNF1B signal density. **c** Volcano plot for DEGs in HNF1B overexpression DU145 RNA-seq. Plots upon the line means *P* < 0.05. Right panel: the overlap of ChIP-seq peaks and DEGs. **d** KEGG analysis of DEGs upon HNF1B overexpression in DU145 cells using the DAVID program. **e** A representative snapshot of HNF1B ChIP-seq and RNA-seq signals located in SLUG gene locus. **f** RT-qPCR analyses of the mRNA levels of CDH1, SNAIL, SLUG, TWIST, ZEB1, and ZEB2 genes in the DU145 cells with a control vector or HNF1B overexpression

HNF1B was previously reported as a transcription factor involved in various developmental processes [13], we next carried out the ChIP-seq assay to determine HNF1B-binding sites in genome. Due to a lack of ChIP-grade antibody, we took the advantage of a DU145 cell line stably expressing HA tagged HNF1B and used HA antibody for ChIP-seq analysis. This experiment yielded a total of 6311 peaks covering 4461 genes, and significantly, 2977 binding events occurred in the promoter regions (transcription start site (TSS) ± 2000 bp). Further analyses found that HNF1B-binding events were significantly enriched at promoter (17.6% versus 1.8% as genome control) and coding exon (5.5% versus 1.9% as genome control) regions of its target genes (Fig. 3a). Heatmap and signal plot analyses also showed that HNF1B bound primarily the TSS and 5′ end regions of its target genes (Fig. 3b).

### HNF1B regulated genes are enriched for cell migration and adhesion functions

To further characterize HNF1B function in regulating its target genes, we performed RNA-seq for the stable DU145 cell lines with either HNF1B overexpression or an empty vector as a control. From the excise, we identified 876 genes that were differentially expressed (| log_2_ (fold change) | > 1, Fig. 3c). Among these 876 differentially expressed genes (DEGs), 335 were bound by HNF1B at either TSS or gene body regions (Fig. 3c), indicating a direct mode of regulation. Moreover, 608 of the 876 DEGs were upregulated on HNF1B overexpressing, while 268 were downregulated, and HNF1B showed similar promoter-binding densities between the two groups (Table S1, Fig. S2), indicating a dual function of HNF1B in activating and repressing target gene expression. Gene ontology (GO) and KEGG analyses of the DEGs showed preferential enrichment for gene sets related to cell movement and adhesion, such as focal adhesion (Fig. 3d), consistent with the results from our phenotypic analyses (Fig. 2b, c).

### HNF1B inhibits prostate cancer cell migration by inhibiting EMT via direct suppression of SLUG expression

To further address the underlining mechanism of HNF1B in suppressing cell migration, we explored the DEGs directly bound by HNF1B and with reported function in regulating cell migration. Such criteria led us to focus on SLUG, whose TSS was bound by HNF1B and whose expression was significantly downregulated upon HNF1B overexpression identified by ChIP-seq and RNA-seq, respectively (Fig. 3e). The reduction of expression was further validated by RT-qPCR (Fig. 3f). SLUG is a classic EMT regulator, and through repressing E-cadherin (CDH1), it plays a critical role in promoting EMT-related cellular behaviors, such as migration and invasion [31]. We thus hypothesized that HNF1B inhibits prostate cancer tumorigenesis by suppression the EMT capability through repressing SLUG. Consistent with the idea, both RNA-seq and RT-qPCR data showed a significantly elevated level of CDH1 upon HNF1B overexpression. Other EMT regulators, did not show detectable changes, except for SNAI1 which showed a moderate reduction upon HNF1B overexpression, suggesting regulation of SLUG by HNF1B is rather specific (Fig. 3f).

**Fig. 4:**
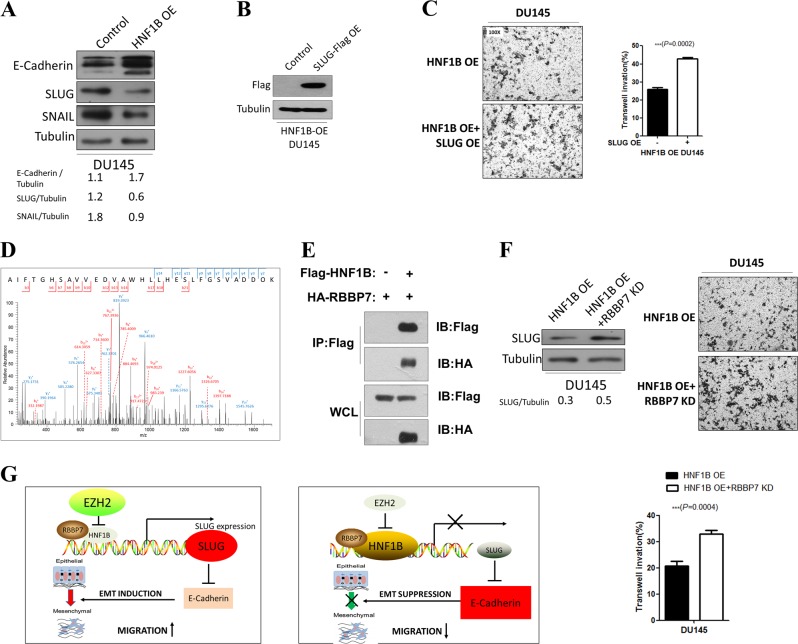
HNF1B suppresses EMT process through repressing SLUG expression with RBBP7. **a** Western blot analyses of EMT markers in the control and HNF1B overexpressing DU145 cells. **b** Western blot analyses of SLUG overexpression in DU145 cells with HNF1B overexpressing. **c** SLUG overexpression rescued HNF1B-mediated reduced cell invasion. **d** Mass spectrometry identification of RBBP7 peptide AIFTGHSAVVEDVAWHLLHESLFGSVADDOK ([M + H]4+ ion at m/z 845.43). **e**. co-IP analysis of the interaction between HNF1B and RBBP7. **f** RBBP7 knockdown induced SLUG upregulation and promoted migration in DU145 cells. **g** Working model depicting the mechanism of EZH2-mediated HNF1B repression and HNF1B/RBBP7-mediated SLUG suppression in prostate cancer

Moreover, the mRNA level changes of SLUG, CDH1, and SNAI1 were also readily confirmed at protein levels (Fig. 4a). To further pin down the role of SLUG in HNF1B-mediated tumor suppressive function, we reintroduced SLUG (Fig. 4b) to examine whether the tumorigenic ability could be restored in HNF1B overexpressed cells. Consistent with the idea, both the migration and invasion abilities of the DU145 cells with HNF1B overexpression were significantly enhanced upon SLUG overexpression (Fig. 4c).

### HNF1B suppresses SLUG by interacting with RBBP7

To gain more insights on how HNF1B might mediate transcription repression, we performed Flag immunoprecipitation followed by mass spectrometry analyses in a stable DU145 cell line with ectopic Flag-HNF1B expression, to identify HNF1B interacting proteins (Table S2). From the potential interacting proteins, we focused on RBBP7. RBBP7 is an ubiquitously expressed nuclear protein and belongs to a highly conserved subfamily of WD-repeat proteins, which is a common component of several transcription corepressor complexes, such as PRC2 and NuRD, and has also been shown to repress EMT-related markers [32]. Using the co-immunoprecipitation (co-IP) assay, the interaction between Flag-HNF1B and HA-RBBP7 were also confirmed in HEK293T cells (Fig. 4e). Importantly, we found that RBBP7 was required for HNF1B-mediated repression of SLUG expression in DU145 cells, as RBBP7 knockdown resulted in 1.6-fold increase of SLUG expression and enhanced migration even when HNF1B was overexpressed (Fig. 4f). And consistently, overexpression of RBBP7 led to significant suppression of SLUG1 and EMT in DU145 cells (Fig. S1C). Taken together, these findings uncovered critical tumor suppressive roles of HNF1B, acting together with RBBP7 to suppress EMT process by inhibiting SLUG expression, and that this pathway is subjected to a negative regulation of EZH2 in prostate cancer cell lines, PC-3 and DU145, and likely in prostate cancer as well.

## Discussion

Metastatic prostate cancer and the development of CRPC are the major catalysts of mortality in prostate cancer patients [33], however the underlying mechanisms are still not clear. It is known that EZH2 is crucial in promoting EMT, a process that is connected to metastasis, and its overexpression has been reported in both metastatic prostate cancer, NEPC and CRPC [34]. Until now, only a few target genes have been studied in depth to clarify the regulatory network of EZH2 in prostate cancer, including CDH1 [34], DAB2IP [35], ADRB2 [36]. Therefore, further understanding of the regulatory network of EZH2 in prostate cancer is still urgently needed. Here in this study, we find that the tumor suppressor HNF1B is repressed by EZH2 in prostate cancer cell lines, and functions together with RBBP7 in suppressing EMT through down-regulation of SLUG expression (Fig. 4g), which expands our knowledge of how EZH2 is involved in this deleterious disease.

HNF1B is part of a large group of transcription factors called homeobox domain proteins. It is found in many organs and tissues, including liver, kidney, reproductive system and urinary tract, and is required for embryonic development. Until recently, HNF1B was reported to function as a tumor suppressor in renal, ovarian, endometrial, colorectal, breast, and prostate cancers. HNF1B loss leads to abnormal regulation of TP53 and RB1 and induction of aneuploidy in renal cancer [37, 38], however, the underlining mechanism in prostate cancer is still not quite clear [22]. Here, our data show that HNF1B directly represses SLUG in mediating its tumor suppressive role and establish a molecular link between EZH2 and EMT process. These findings are of importance, as EZH2 has been previously connected to EMT process via direct or indirect regulation of E-cadherin/CDH1 [34]. Now our study provides a new downstream path of EZH2 leading to EMT via tumor suppressor HNF1B.

HNF1B has been reported to frequently undergo silencing in various cancer types [37, 38]. Recent researches revealed that DNA methylation mediated HNF1B silencing frequently occurs in many cancers [39]. In ovarian cancer, methylation was detected in the region covering CpGs 16-19 of HNF1B gene in over 60% tumor samples while none was found in control samples [40]. Previous study in prostate cancer also showed that HNF1B promoter methylation correlates with prostate cancer risk SNP genotype and HNF1B expression [22]. In this study, we uncovered an alternative repression mechanism of HNF1B through EZH2-mediated histone H3K27me3. Our findings elucidate a previously underappreciated path of EZH2 exerting its oncogenic effects through repressing HNF1B and promoting SLUG mediated EMT process in prostate cancer. Beyond prostate cancer cell lines, we also observed a significant reverse correlation between EZH2 and HNF1B expression in patient samples, and the high EZH2 and low HNF1B pattern is strongly associated with poor prognoses and advanced metastatic state (our own collection and public database). Thus, our results provide for the first line evidence of EZH2-mediated repression of HNF1B during prostate cancer progression, which may also exist in other cancer types. Interestingly, three *HNF1B* associated SNPs, rs11649743, rs4430796, and rs7501939, occurring in the intronic regions of *HNF1B*, were associated with decreased risk of prostate cancer by GWAS. In light of our findings, it will be intriguing for future investigation of whether these SNPs may interfere the EZH2-mediated silencing mechanisms thus leading to enhanced expression of HNF1B.

Our study for the first time determined the role of RBBP7 as a corepressor associated with the tumor suppressor HNF1B to repress SLUG expression and EMT phenotype in prostate cancer. RBBP7 is a cofactor of several chromatin repressor complexes, and has been reported to exert either promoting or repressing function on EMT in a context dependent manner. RBBP7 has also been reported to suppress E-cadherin/CDH1 by interacting with TWIST and recruiting the complex to proximal regions of the E-cadherin promoter, thus inducing EMT [41]. In addition, RBBP7 also interacts with NKX6.1 to directly repress Vimentin via EZH2-mediated H3K27me3 and induces EMT progress [32]. On the other hand, in lung cancer cells, RBBP7 acts as a transcriptional activator of the E-cadherin/CDH1 gene by binding to its promoter region thereby repressing EMT progress [42]. Ours and together with other’s findings reveal a context dependent manner of RBBP7 function in the regulation of EMT. Importantly, our study clearly demonstrates a tumor suppressive function of RBBP7 in prostate cancers when HNF1B is present.

EZH2 inhibitor has been extensively explored in various cancers recently, while its implication in prostate cancer has been controversial [43, 44]. Here, the demonstration of a critical axis of EZH2-HNF1B/RBBP7-SLUG in prostate cancer development, provides mechanistic insights of EZH2-mediated prostate tumorigenesis and molecular basis for the application of EZH2 inhibitors. Although the study was based on RNAi and overexpression approaches, our findings call for future investigation of EZH2 inhibitors in treating tumors, including prostate cancer, with low *HNF1B* expression mediated by EZH2 overexpression.

## Materials and methods

### Constructs

pPB-CAG-EBNXN vector was a kind gift from Sanger Institute. pPB-CAG-ires-Pac was generated as previously described [45, 46]. pPB-CAG-HNF1B-ires-Pac, pPB-CAG-EZH2-ires-Pac, and pPB-CAG-SLUG-ires-Pac were generated by ligating full length HNF1B, EZH2, and SLUG into the multiple-cloning sites of pPB-CAG-ires-Pac.

### Cell lines and cell culture

LNCaP, PC-3, and DU145 cells were obtained from ATCC (Bethesda, MD, USA) and maintained in RPMI-1640 supplemented with 10% FBS and antibiotics (100 units/ml penicillin and 0.1 mg/ml streptomycin), and grown at 37 °C in standard cell culture conditions (5% CO_2_, 95% humidity). Control, HNF1B, RBBP7, or EZH2 overexpression stable cells were obtained by co-transfection of pPB-CAG-ires-Pac, pPB-CAG-HNF1B-ires-Pac, pPB-CAG-RBBP7-ires-Pac, or pPB-CAG-EZH2-ires-Pac with pCMV-PBase. After 2 μg/ml puromycin (Amresco) screening for 2 weeks, stable cell lines were selected and identified by western blotting. SLUG overexpression in HNF1B-DU145 cells were obtained by transfection of HNF1B-DU145 cells with pPB-CAG-SLUG-ires-Pac. All cell lines were authenticated by STR profiling and tested for mycoplasma contamination.

### siRNA transfections

In this study, we tested the individual set of three siRNAs (GenePharma, China) against HNF1B by western blotting. The effective single siRNAs (GenePharma, China) against HNF1B were used for further experiments. The knockdown of EZH2 was accomplished with two different siRNA duplex (GenePharma, China) as described earlier [10].

For siRNA transfection in sixwell plates, 3 × 10^5^ cells per well were subjected to reverse transfection with 20 nM siRNA (GenePharma, China) using Lipofectamine 2000 transfection reagent (Invitrogen), following the manufacturer’s instructions.

### Antibodies and immunoblotting

Cells were lysed using 1× SDS loading buffer (50 mM Tris-HCl pH 6.8, 10% glycerol, 2% SDS, 0.05% bromophenol blue, and 1% 2-mercaptoethanol). Antibodies were listed as follows: anti-HNF1B antibody (12533-1-AP, Proteintech), anti-E-cadherin (610181, BD Transduction Laboratories), anti-SLUG (C19G7, Cell Signaling Technology), anti-SNAI1 (C15D3, Cell Signaling Technology), anti-EZH2 (AC22, Cell Signaling Technology), anti-HA (51064-2-AP, Proteintech), anti-FLAG (20543-1-AP, Proteintech), anti-RBBP7 (20365-1-AP, Proteintech), and anti-tubulin (ab134185, Abcam). For immunoblot, proteins were separated by SDS–PAGE and transferred to polyvinylidene difluoride membranes (Millipore). HRP-conjugated secondary antibodies (Jackson laboratories) and enhanced chemiluminescence system was used for signal detection. Protein was visualized using KODAK film machine or ChemiDoc XRS chemiluminescence detection and imaging system (Bio-Rad Laboratories).

### Cell proliferation (MTS) assay

The MTS assay was performed as described elsewhere [47]. In brief, cells were seeded at 4000 cells/well (0.1 ml) in 96-well plates and incubated overnight at 37 °C for 6 days. At the end of the experiments, 20 μl of CellTiter 96 AQueous One solution reagent MTS (Promega) in 100 μl of RPMI-1640 were added to each well, incubating cells for 1 h. Cell viability was estimated by monitoring the absorbance at 490 nm using a SYNERGY HT microtiter plate reader (Bio-tek).

### In vivo tumor growth assay

Six-week-old male athymic mice were inoculated s.c. with 100 μl of a mixture containing 1 × 10^7^ prostate cancer cells. Tumor growth was monitored weekly, and mice were sacrificed after 4 weeks. Tumor sizes were measured recorded in mm^3^ (length × width^2^). There were eight mice in each group. The Ethics Committee of Nanjing Medical University approved all animal use procedures.

### Cell migration assays

Cell migration assays were carried out in 24-well tissue culture plates with Transwell inserts (Corning) as described elsewhere [47].

### Wound-healing assay

Cells spread within 12-well dishes at 1 × 10^5^ cells/well and cultivated till full confluence. Cell monolayers were removed by a sterile white micropipette, which resulted in a denuded area with a fixed width. Phosphate buffered saline (PBS) was used to wash off cell debris, and then, culture medium was added to the cell culture. During the indicated period after being wounded, wound closure was monitored, and photographed.

### Real-time RT-PCR

Total RNAs were extracted from cells using TRIzol reagent (Invitrogen). RNA was subjected to reverse transcription with reverse transcriptase as Manufacturer’s instructions (Fermentas). Quantitative real-time PCR was performed using the Bio-Rad CFX96 system, and the relative gene expression was normalized to GAPDH as a control. Table S3 showed primer sequences used in the study.

### ChIP-qPCR and ChIP-Seq

ChIP assays were carried out as previously described [48]. Briefly, DU145 were crosslinked with 1% formaldehyde for 10 min at room temperature followed by quenching with 0.125 M glycine. After washing by PBS twice, the crosslinked cells were suspended in ChIP lysis buffer (50 mM HEPES-NaOH pH 7.5, 500 mM NaCl, 1 mM EDTA, 1% Triton, 0.05% SDS, 0.1% sodium deoxycholate with freshly added proteinase inhibitor, and PMSF) and the chromatin sizes were sonicated to 200–300 bp. For HA-HNF1B ChIP, the chromatin was incubated with HA antibody (3724S, Cell Signaling Technology) overnight at 4 °C, and additionally with prewashed protein A/G agarose beads (SA032005, Smart-lifesciences) for 1 h. The beads were washed three times with the lysis buffer, twice with low salt buffer (10 mM Tris-HCl pH 8.0, 250 mM LiCl, 1 mM EDTA, 0.5% NP-40, and 0.5% sodium deoxycholate), and once with TE (10 mM Tris-HCl pH 8.0, and 2 mM EDTA). Elution and reverse crosslinking were then carried out in the elution buffer (50 mM Tris-HCl pH 8.0, 10 mM EDTA, and 1% SDS) at 65 °C for 4 h. After 1 h digestion at 55 °C with RNase A and Proteinase K, DNA samples were purified using the PCR purification kit (QIAGEN). The DNA samples were analyzed using real-time PCR and prepared for deep sequencing by Accel-NGS 2S Plus (Swift, 21096) and sequenced by Illumina HiSeq X Ten (BasePair Biotech. Co., Ltd, Suzhou). Heatmaps and signal plots were generated with ngs.plot [49].

### RNA-seq and analysis

RNA-seq was carried out according to manufacturer’s guidelines (C-10365; Life Technologies) and previous study [50]. GO analysis and KEGG analysis were carried out using the database for annotation, visualization, and integrated discovery (DAVID) website [51]. Data of RNA-seq were provided in Table S2 in Supplementary information.

### IP-mass spectrometry

Cells were harvested with EBC lysis buffer (50 mM Tris-HCl, pH 8.0, 120 mM NaCl, and 0.5% Nonidet P-40) supplemented with protease inhibitors (Selleck Chemicals) and phosphatase inhibitors (Selleck Chemicals). For IP, 800 μg of cell lysates were incubated with the flag antibody (1–2 µg) for 3 h at 4 °C, and then protein A-Sepharose beads (GE Healthcare) were added into a mixture of cell lysates and antibody, and then incubated at 4 °C for another 1 h. IP complexes were washed five times with NETN buffer (20 mM Tris-HCl, pH 8.0, 100 mM NaCl, 1 mM EDTA, and 0.5% Nonidet P-40). After washing, the IP samples were resolved by SDS–PAGE on a 4–20% polyacrylamide gel (Bio-Rad) and visualized using the Bio-Safe Coomassie Stain (Bio-Rad). The gel containing FLAG-HNF1B complex was excised and treated with dithiothreitol to reduce disulfide bonds and iodoacetamide to alkylate cysteines. In-gel digestion of the protein was performed with trypsin or chymotrypsin. The resulting peptides were extracted from the gel and analyzed by liquid chromatography tandem mass spectrometry. All peptide matches were filtered on the basis of mass accuracy, tryptic state (for trypsin), and XCorr, and confirmed by manual inspection. The mass spectrometry proteomics data generated in our study have been provided in Table S3 in Supplementary information.

### Publicly available gene expression datasets and clinical datasets

GSE21032, GSE35988, GDS1439, GSE16560, and a group of similar prostate adenocarcinoma samples at the cBioPortal for Cancer Genomics (The Cancer Genome Atlas (TCGA), Provisional) were used in this study [52].

### Analyzing correlation between the HNF1B/EZH2 expression and risk of biochemical recurrence

For analyzing the correlation between the expression of HNF1B/EZH2 and the risk of biochemical recurrence, we used an array of primary prostate adenocarcinoma samples with EZH2 and HNF1B expression data from TCGA collection, and the clinical variables were acquired from the cBioPortal for Cancer Genomics. Seventeen Samples in the datasets were separated into four groups according to HNF1B and EZH2 expression. Survival curves were generated using the GraphPad 5.0.

### Statistical analyses

The results were presented as the average values ± standard error of mean (SEM). By the use of Kaplan–Meier method with log-rank test, overall survival rates could be actuarially calculated and evaluated from the day after surgery. Between-group variations were evaluated by the use of the *χ*2 test, Student’s *t* test, the Mann–Whitney *U* test, and repeated-measures analysis of variance (ANOVA) test. Spearman’s correlation finished the exploration of the relationships. For mice studies, sample size was determined using the methods as previously described [53]. A *p* value below 0.05 was thought to have statistical significance. Predetermined exclusion criteria included the absence of signal at the start of the experiment. *P < 0.05, **P < 0.01, ***P < 0.001.

## Supplementary information

Supplementary legends

Table s1

Table s2

Table s3

Sup figure s1

Sup figure s2
