# Supplementary material for: HNF1B-mediated repression of SLUG is suppressed by EZH2 in aggressive prostate cancer
Source: Oncogene. 2019 Oct 21;39(6):1335–46. doi: 10.1038/s41388-019-1065-2 (PMC7002300; doi:10.1038/s41388-019-1065-2)
Supplement: Supplementary file 1 — Supplementary legends [file 41388_2019_1065_MOESM1_ESM.docx]

Supplementary Figure S1.

A. EZH2 mRNA level in metastatic prostate cancer (GSE21032 and GDS1439).

B. Inverse correlation between SLUG and HNF1B mRNA expression in prostate samples from Fred Hutchinson CRC and GSE21032.

C. RBBP7 overexpression in DU145 cell induced SLUG downregulation and reduced cell migration.

Supplementary Figure S2.

HNF1B showed similar promoter-binding densities between the up-regulated and down-regulated genes.

Supplementary Table S1 List of upregulated and downregulated genes in RNA-seq.

Supplementary Table S2 List of HNF1B interacting proteins identified by mass spectrometry.

Supplementary Table S3 List of the oligo sequences of PCR primers used in this study.
