# Supplementary figures and images for: HNF1B-mediated repression of SLUG is suppressed by EZH2 in aggressive prostate cancer
Source: Oncogene. 2019 Oct 21;39(6):1335–46. doi: 10.1038/s41388-019-1065-2 (PMC7002300; doi:10.1038/s41388-019-1065-2)

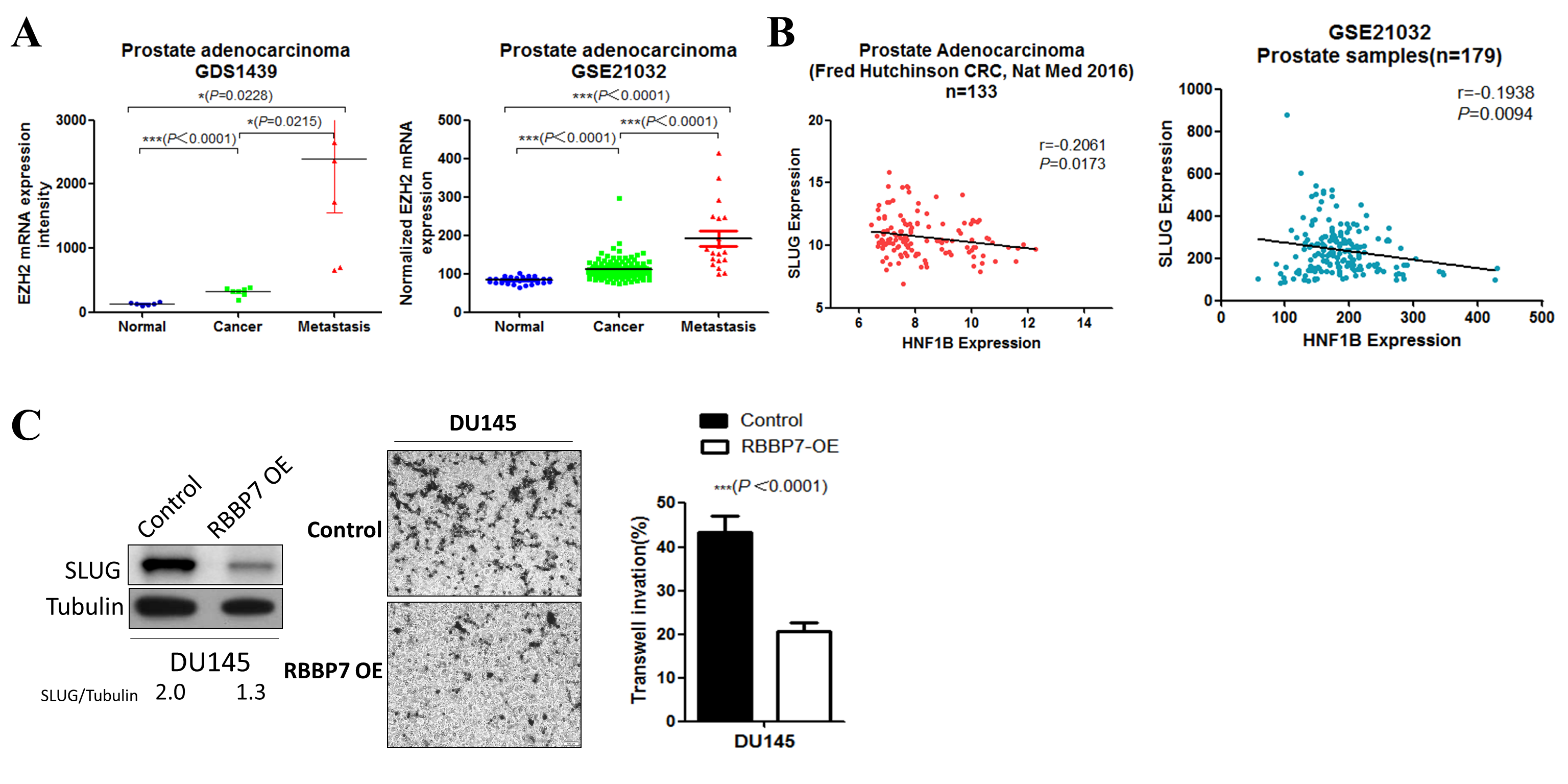

Supplement: Supplementary file 5 — Sup figure s1 [file 41388_2019_1065_MOESM5_ESM.tif]

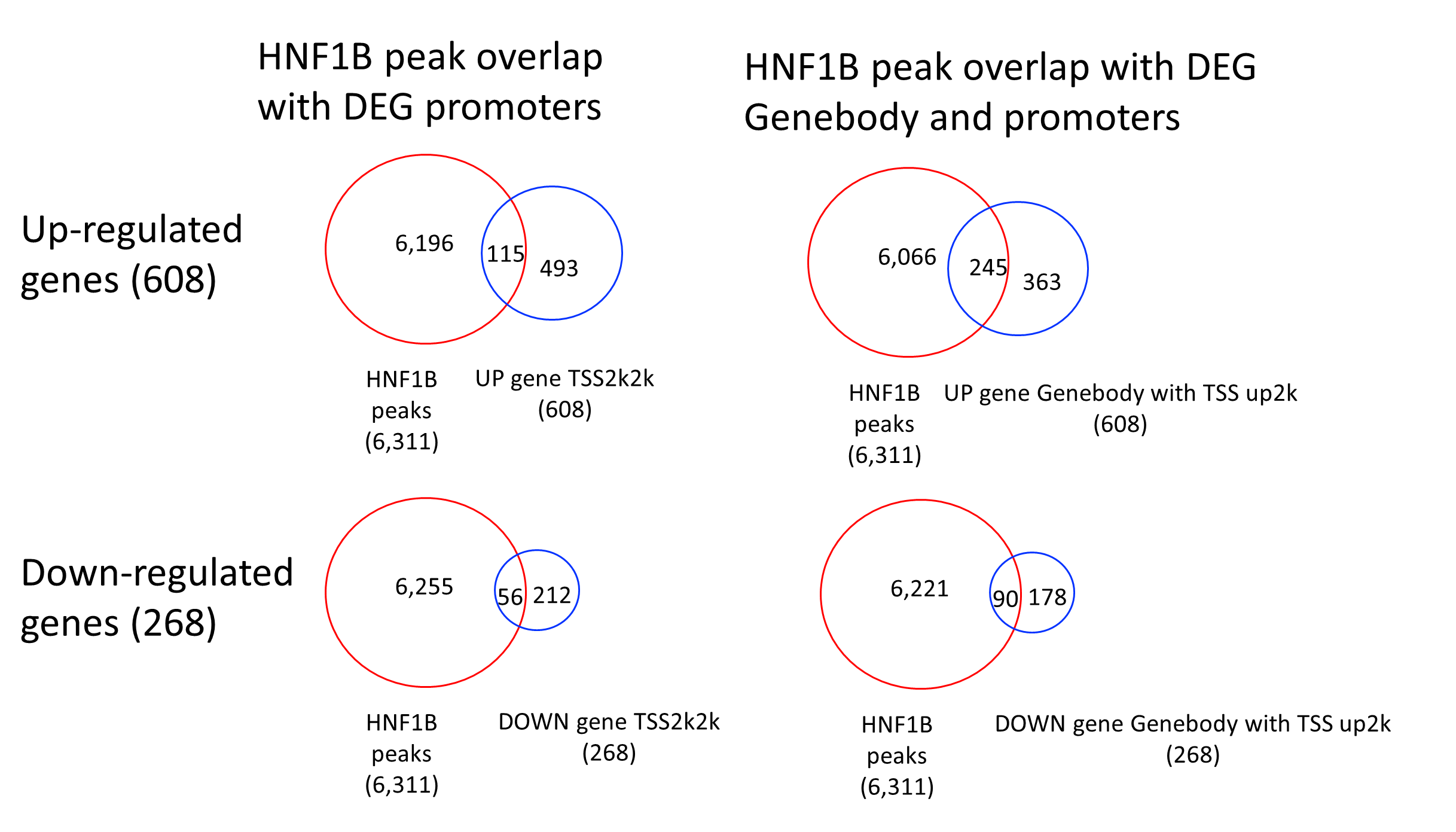

Supplement: Supplementary file 6 — Sup figure s2 [file 41388_2019_1065_MOESM6_ESM.tif]
